# How Parents Perceive Their Children’s Body Weight: Insights from a Sample of Schoolchildren from Vienna, Austria

**DOI:** 10.3390/nu16234094

**Published:** 2024-11-27

**Authors:** Paula Moliterno, Stefanie Franceschini, Victoria Donhauser, Kurt Widhalm

**Affiliations:** 1 Austrian Academic Institute for Clinical Nutrition, 1090 Vienna, Austria; paula.moliterno@eddykids.at (P.M.);; 2 Division of Clinical Nutrition and Prevention, Department of Pediatrics, Medical University of Vienna, 1090 Vienna, Austria

**Keywords:** parental perception, childhood obesity, child’s weight perception, school-based intervention, Austria

## Abstract

Background/Objectives: Effective school-based childhood obesity prevention strategies should include parental involvement. In the EDDY (“Effect of sports and diet training to prevent obesity and secondary diseases and to influence young children’s lifestyle”) program, we aimed to describe parental perceptions of children’s body weight in a sample of schoolchildren from Vienna, Austria. Methods: A cross-sectional analysis, including 128 children from the third grade in three Viennese primary schools, was conducted. A self-administered questionnaire was used to collect sociodemographic data and parentally reported weight and height. Parental perception of the child’s weight status was assessed using the question, “In your opinion, you would describe your child as being”. Children’s nutritional status was assessed through measurements. Results: Almost 15% of the children had obesity. The median SDS-BMI was 0.39 (−1.00–2.83) and 0.21 (−1.39–2.47) for girls and boys, respectively. More mothers of girls had overweight/obesity compared to mothers of boys (59.2% vs. 41.1%, *p* = 0.05). Almost half (48.4%) of the parents underestimated their child’s weight. The percentage of mothers with overweight/obesity was higher in the group that underestimated their child’s weight (59.3% vs. 37.7%, *p* = 0.018). Parents perceived 59.4% of the children as having a normal weight, while BMI classification indicated that 71.9% had a normal weight. Misperception was higher among children who were overweight (75%) and obese (89.5%). Parents of children with a normal weight were less likely to underestimate [Adjusted OR = 0.16 (95% CI: 0.050–0.49)]. Conclusions: The prevalence of misperception was high, especially for children with overweight or obesity. These findings highlight the need to incorporate children’s adequate weight recognition into the EDDY program as part of parents’ content.

## 1. Introduction

Current childhood overweight and obesity rates in 8-year-old children in Austria show that one in four boys and almost one in four girls [1] are affected by this public health problem. During the last few years, an increase in obesity rates in school-aged children in Vienna has been reported [2]. Recognizing how parents/caregivers perceive their children’s weight status is vital in tackling childhood obesity, as a first step is recognizing excessive body weight [3]. A few systematic reviews have reported a high percentage of parents who misperceive their child’s body weight, especially in the case of parents of children who are overweight or obese [4,5,6]. Previous findings in a meta-analysis [7] were recently confirmed by Alshahrani et al., reporting that 55% (95% CI 49–61%) of parents underestimated their child’s level of overweight and obesity [8]. Children are highly dependent on their parents’ behavior, particularly at school age, and previous reports have shown that misperception of weight status is associated with younger age [5,6]. If parents misjudge their child’s weight, they might not see the need to adopt healthier habits when needed. Moreover, they may also need more motivation to pursue healthy choices in everyday life. On the other hand, for many parents or caregivers, the topic of their children’s weight may lead to resistance and negative emotions. It has been described that the medical terms that health professionals often use do not agree with those commonly used by parents to refer to excessive weight [8], adding difficulty to communication when trying to align efforts for a behavior change. Many parents have a limited understanding of how excessive weight is defined, and they usually do not use or trust objective measures when identifying overweight in children [9]. Acknowledging this and describing parental perception of body weight can foster better support for healthy behaviors when planning educational interventions in the school setting [10]. As excessive body weight is common nowadays, it may become “normal”, challenging parents’ ability to identify a healthy body size [11]. Parents play a significant role in supporting their young children’s health. Therefore, involvement in any strategy to promote changes for a better quality of life must consider their previous knowledge and perceptions of a healthy body weight [10]. For example, when analyzing the parents’ perception of the causes of excessive body weight, a study found that the inherited factor appeared dominant [5]. Moreover, parents of children with overweight and obesity reported more school-related problems, lower emotional functioning, and overall poorer health [12]. Acknowledging those important aspects for parents is crucial in planning tailored interventions, like the EDDY (“Effect of sports and diet training to prevent obesity and secondary diseases and to influence young children’s lifestyle”) program implemented in some schools in Vienna, Austria. Until now, no thorough situational analysis regarding parental perception of body weight status has been considered in the EDDY’s time planning. Moreover, information regarding parental perception in Austria is limited, with only one up-to-date study reporting, from a general pediatric outpatient clinic in Vienna, a high parental weight status misclassification [13]. This study aimed to describe parental perceptions of their children’s body weight status in a sample of schoolchildren from Vienna, Austria, and to study associations with current nutritional status, assessed by objective measures of sex- and age-specific body mass index (BMI).

## 2. Materials and Methods

### 2.1. Study Sample

A non-randomized sample of third-grade students from three schools in Vienna, Austria, who were willing to participate in the EDDY (“Effect of sports and diet training to prevent obesity and secondary diseases and to influence young children’s lifestyle”) program, were included. The three elementary schools included were selected by the Federal Ministry of Education, Science and Research of Austria and were located in the 23rd, 12th, and 10th districts. Regarding the districts’ socioeconomic status, the 23rd district boasts an average net income slightly above the city and national averages [14]. In contrast, the 10th district ranks among those with lower average incomes, and the 12th district is within the mid-range, with an average annual net income that remains below the overall average. Regarding the percentage of unemployed individuals, the 23rd district performs better than the Vienna average, while the 10th and 12th districts face higher unemployment rates than the city average. The 23rd district has a relatively low proportion of individuals with academic degrees compared to other Vienna districts but stands out with one of the highest percentages of residents holding vocational training qualifications. Meanwhile, the 10th and the 12th show fewer individuals with academic degrees than other districts in Vienna [14]. All children with complete anthropometric data and parent questionnaire information at baseline were selected. After excluding n = 9 children without information on parental body weight perception and n = 1 children without anthropometric data, we finally included n = 128 children with complete data on outcome variables.

### 2.2. Ethics

Approval for the study’s ethical considerations was obtained from the Ethical Committee of Sigmund Freud University, Vienna (PAFGRW9O@EFQV885378—15 September 2016). Parents provided written consent for their child’s participation in this study. Moreover, older children also gave written assent for their participation. All data collected underwent an anonymization process. Participation was voluntary, and no compensation was provided. The ability to drop out was allowed at any time.

### 2.3. Parental Perception of Child’s Weight Status

Parental perception of children’s weight status was collected from a self-administered questionnaire in the German language at the beginning of the school year in October 2022, using the question, “In your opinion, you would describe your child as being…”. A scale with five response categories was selected (very underweight, a little underweight, the right weight, a little overweight, very overweight). To study the concordance between parental perceived weight status and the child’s actual weight status by BMI category, the original categories of ‘very underweight’ and ‘little underweight’ of parental perception were merged into one ‘underweight’ category. Later, the parent’s assessment was assessed and considered accurate if children with low weight were correctly classified as “underweight”, normal weight children as “the right weight”, children with overweight as “a little overweight”, and children with obesity or extreme obesity as “very overweight”. We further estimated parental misperception and classified it based on if parents underestimated, agreed with, or overestimated the child’s weight status. Underestimation was defined as a parental perception lower than the child’s actual weight status as defined by the BMI category classification (see Section 2.4). In comparison, overestimation was defined as a parental perception higher than the child’s current weight status as defined by BMI classification. A parent was classified as in agreement if the child’s weight status was the same according to objective BMI classification.

### 2.4. The Child’s Real Weight Status

Children’s body weight was measured using a Tanita body composition electronic scale (MC-780MA, TANITA Corporation, Tokyo, Japan), and height was measured using a stadiometer (SECA 213, Hamburg, Germany), with the child standing without shoes. After body weight and height were measured, BMI (kg/m^2^) was calculated and transformed to age- and sex-specific percentiles to classify nutritional status [15]. Low weight was classified as being in the <3rd percentile, normal weight in the ≥3rd percentile, and overweight in the ≥90th percentile. The obesity category was defined as the sum of the original categories of obesity (≥97th percentile) and extreme obesity (≥99.5th percentile) [15]. As only one child was classified as low weight, we combined the low with the normal weight category. The BMI-SDS was calculated using the LMS method [15].

### 2.5. Parental Weight Status and Additional Information

Parents were asked to report their weight (kg) and height (cm). Further, BMI (kg/m^2^) was calculated. Mothers’ nutritional statuses were also classified into a binary category: “non-excessive weight” if BMI was ≤24.9 kg/m^2^ and “excessive weight” if BMI was ≥25 kg/m^2^.

Additionally, data on age, nationality, the education level of the parents, household monthly income, and the number of siblings were collected from the self-administered questionnaires.

Parents’ level of education was assessed through a categorical question that included six options, ranging from “no formal education” to “13 or more years of education (University degree)”. For analysis, the variable education level was transformed into four categories: “≤4 years”, which included no formal education or primary school only, “5–9 years”, “10−13 years”, and “≥13 years”. To study parental misperception, the mother’s level of education was classified into a binary category: “<13 years” and “≥13 years”.

The monthly household income question asked about all family members’ income, including family allowance. The answer options included a list of 13 categories from “<500 Euro” to “≥5000 Euro”. Further, the variable was transformed into four categories: “<1500 Euro”, “≥1500 <2500”, “≥2500 <5000”, and “≥5000 Euro”.

### 2.6. Statistical Analysis

Data distribution was checked for normality using the Shapiro–Wilk test. Since data were found to not be normally distributed, nonparametric analyses were conducted. Descriptive analyses were performed by calculating medians, 95% confidence intervals (CI), and frequency distribution. Parents’ perception of their children’s weight status was analyzed using cross-tabulation according to their children’s real weight status. The kappa index was used to evaluate the agreement between the parental perception of weight status and real BMI classification, following the cutoff points proposed by Landis and Koch to describe the strength of agreement [16]. The results of participant’s characteristics by parental misperception of child’s weight status (underestimation, agreement, or overestimation) were presented as numbers (percentages) for categorical variables and as medians (95% CI) for quantitative variables. We compared underestimation vs. agreement and overestimation vs. agreement using Wilcoxon’s *t*-test. The correlation between the child and mother’s BMI was assessed using the Spearman correlation test, adjusted by school, sex, and age.

Logistic regression analysis was performed to calculate unadjusted (OR) and adjusted odds ratios (aOR) and a 95% CI for the parental underestimation of child weight status. Considering the small sample size [17], candidate variables to be included in the model were selected using a literature review. Then, we followed a variable selection method that began by performing a bivariate analysis of each variable, selecting the set of variables that obtained a *p*-value ≤ 0.20 for its use in building multivariate logistic regression models [17]. The latter included the categorical variables of maternal nutritional status, the child’s nutritional status, maternal age, and household income as control variables in the multivariate logistic regression models. Categorical variables, instead of their continuous counterparts (e.g., nutritional status classification by BMI instead of continuous BMI), were preferred to avoid potential collinearity and enhance the results’ explainability. A sensitivity analysis was performed to evaluate the impact of including either a categorical variable or its continuous counterpart in the models. Additionally, models were adjusted for the school group to account for any differences in the school environment. As only two observations were registered for the parental overestimation of child weight status, the OR was not estimated.

Data analysis was conducted using SAS OnDemand for Academics (Cary, NC, USA), and a *p*-value < 0.050 was assigned as significant.

## 3. Results

The median age of the participants was 7.9 (7.2–9.3) years, with no difference observed between the sexes (Table 1). Of the children, n = 45, n = 49, and n = 34 belonged to schools 1, 2, and 3, respectively. Between schools, no differences were observed in the age (*p* = 0.12), SDS-BMI (*p* = 0.19), or nutritional status by BMI classification (*p* = 0.092) of the participants. Table 1 shows anthropometric characteristics, nutritional status classifications, and parents’ perceptions of children’s weight status, among other general characteristics.

The number of girls with obesity was higher than for boys (*p* = 0.011). Moreover, the percentage of mothers with excessive body weight was significantly higher for mothers of girls than for mothers of boys (*p* = 0.05). The median age of mothers was 37 (27–47) years old and of fathers was 39 (32–51) years old, with no difference between the sex of the child (*p* ≤ 0.82). Fifty-two percent of the mothers were 37 years old or younger, and 61.1% of both parents reported being born outside Austria (Table 1).

**Figure 1:**
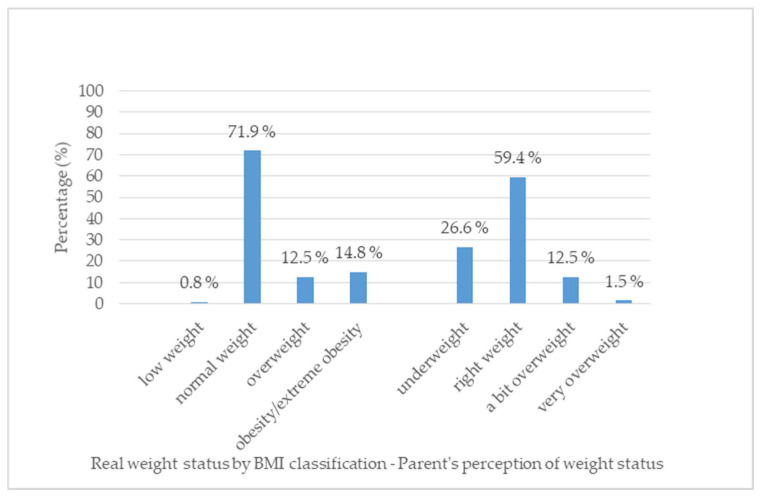
Parent’s perception of their child’s weight status vs. real BMI classification.

Parents were likely to misperceive their children’s weight status: 59.4% reported their children as having a normal weight, while 71.9% of the children were categorized as having a normal weight according to BMI classification. We found that 26.6% of the parents perceived their child’s weight as being lower than it should be, when, according to BMI, only 0.8% of the children were underweight (Figure 1). A fair agreement between perceived weight status and real BMI classification was observed [kappa = 0.28, (95% CI 0.17–0.38), *p* = 0.035].

**Figure 2:**
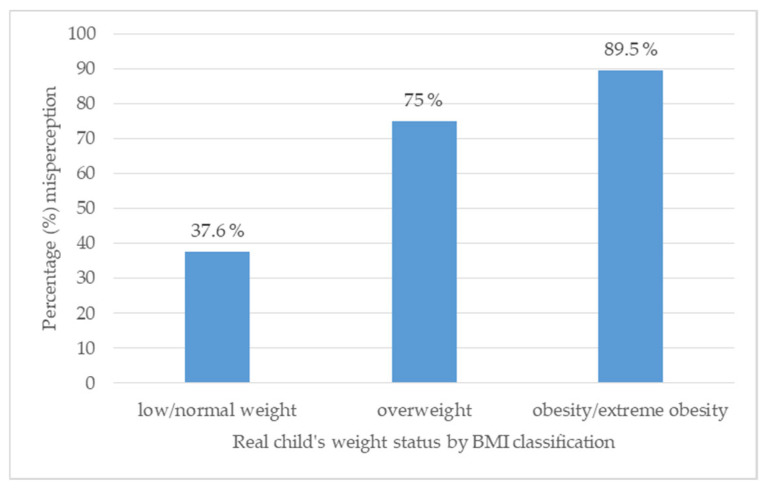
Parental misperception of children’s weight status by actual nutritional status.

Moreover, parents were more likely to misperceive children with obesity or extreme obesity (89.5%) than children who were overweight (75%), or low or normal weight (37.6%). A chi-square test of independence showed that the relationship between the child’s weight status and parental misperception was statistically significant, X^2^ (DF = 2, 21.53), *p* < 0.0001 (Figure 2).

When analyzing the relationship between the child’s and mother’s nutritional status (Figure S1), a positive, low, but significant correlation was observed between the child’s and mother’s BMI in models adjusted by the school, sex, and age variables (*r* = 0.20, *p* = 0.029).

Participants’ characteristics based on the parental misperception of their child’s weight status are shown in Table 2. No differences were observed between the weight status misperception groups for the child’s age and sex distribution.

The parental underestimation of a child’s weight status was not associated with SDS-BMI or BMI. However, the percentage of children with overweight and obesity was higher in the group of parents who underestimated their child’s weight (45.2% vs. 10.9%, *p* < 0.0001) compared to the reference group (agreement) (Table 2). Regarding parental characteristics, underestimating the child’s weight status was not associated with the parent’s age, background, maternal education, or number of siblings. However, the mothers’ BMIs in the group that underestimated the child’s weight were higher than the BMIs of the mothers in the reference group (26.4 kg/m^2^ vs. 23.3 kg/m^2^, *p* = 0.036). Moreover, the percentage of mothers with excessive body weight was higher in the group that underestimated body weight than in the group that accurately perceived it (59.3% vs. 37.7%, *p* = 0.018). A marginal association was observed with household income (*p* = 0.057), with lower reported incomes in the group where parents underestimated the child’s weight compared to the reference group (agreement) (Table 2).

After accounting for the maternal and child’s nutritional status, household income, and school, the parental underestimation of children’s weight was associated with significantly lower odds for children with normal weight [aOR = 0.16 (95% CI: 0.050–0.49)] compared to children with overweight or obesity (Table 3).

## 4. Discussion

In this non-representative sample of school-aged children from Vienna, our results show a high level of misperception of children’s weight status by parents, particularly if the children had overweight or obesity. Parents were 84% less likely to underestimate the weight of children with normal weight compared to those with overweight or obesity. This study aligns with findings from a recent systematic review [5] and previous research from an outpatient pediatric clinic in Vienna [13], indicating that children with overweight are more likely to be misclassified by their parents. Although objectively it would be easier to identify excessive body weight at higher stages of obesity, our study, similar to others [13], found that the higher the BMI, the higher the percentage of parental misperception of their children’s nutritional status. Parents of children with overweight misperceived their children’s weight status by 75%, rising to 89.5% for children with obesity or extreme obesity. These results are consistent with those from a previous systematic review [4] and other studies following the same trend [6,7,9,11], though they reported lower percentages of misperception.

Cultural or economic factors may contribute to these high misperception rates [6]. It has been described that living in an environment where many children have excessive weight can normalize this condition, affecting parental perceptions [11]. In Austria, the prevalence of overweight and obesity remains high, affecting 25% of boys and 23.6% of girls aged 8 [1]. A United Kingdom qualitative study reported that parents often use extreme cases in their child’s school or community as reference points when determining childhood obesity [9].

Cultural ideals around body size also influence these perceptions, with a stronger emphasis on ‘thinness’ expected for girls [18]. In families with an Asian background, overweight in boys is seen positively as sign of prosperity, which may be linked to parental underestimation, as reported recently [19]. This sociocultural difference related to sex may lead parents to classify boys in lower weight categories than girls [20]. However, our study found no significant differences in parental weight perceptions based on the child’s sex. It has been noted that the relationship between cultural background and parental perception of child weight status in Europe needs to be well-documented [20]. In our study, parental misperception was not related to the parents’ country of origin (as an indicator of migration background), likely due to the high percentage of parents with a migration background in our sample, higher than the 46% reported in Vienna [21].

On the other hand, parental perception of weight status was associated with income, another factor closely related to migration background. We observed a trend where the parents who underestimated their child’s weight status reported lower incomes. It has been described that immigrants in Austria often have a lower socioeconomic position compared to locals and a higher prevalence of overweight among women and children [22].

Although parental concern about children becoming overweight has been associated with the child’s BMI, a recent cohort study involving five European countries found that this concern did not influence parental perception of weight status [20]. Nevertheless, parents’ concern about their child’s weight as a health problem has been linked with increased odds of being in a proactive stage of making lifestyle changes for children with overweight or who are at risk for overweight [23]. This highlights the importance of immediate health concerns in motivating some parents to engage in lifestyle interventions, an important fact considering that children with overweight had a higher prevalence of multiple comorbid conditions compared to children with normal weight [12]. Moreover, it reflects how strategies to tackle childhood overweight and obesity must be carefully planned to achieve family involvement and long-term results. These strategies should address parents’ concerns and interests to promote their involvement [10], especially in Austria, where participation rates in studies have proven to be below 50% [1].

In our study, 27.3% of children were classified as being overweight or obese. However, parental perception indicated that only almost half of these children were overweight or obese. In turn, a large proportion (48.4%) of the parents underestimated their child’s weight, indicating that it was lower than in reality. These results are partly consistent with findings from meta-analyses [7,8] and a European cohort study of 8-year-olds [20]. However, our sample showed higher rates of overweight and obesity compared to a previous study with the same age range in Vienna [13], possibly due to the school setting; parents may be more willing to participate in school-based studies than in health institution studies where they may feel judged. In this sense, the EDDY program presents a unique opportunity to address the issue of weight status perception in Vienna, as it integrates educational content into school curricula and works in alignment with teaching staff. Based on the current results and a theoretical framework, this topic can be addressed with parents [24]. The school setting offers a safe space where parents can receive information and support related to healthy weight and lifestyle, contributing to changing the home environment [10].

On the other hand, the high misperception rate in our study may also be influenced by the tool and terms used to assess parental perception of body weight, potentially increasing selection bias [6]. The term “overweight” may seem stigmatizing or offensive to parents [25] or even uncomprehensive [9], lowering their likelihood of selecting that label even when they agree with it. Using a nonvisual scale (as in our case) rather than sketches or silhouettes from a visual scale might have made it harder for parents to label their child’s nutritional status [6] accurately.

The parental misperception of children’s weight has also been linked to parents’ body weight [13,26]. In our study, there was a poor correlation between the mother’s and child’s BMI. Mothers who underestimated their child’s weight had a higher BMI than those who provided accurate assessments, consistent with previous findings [13,26]. Moreover, this connection between children being overweight and maternal overweight has been observed even at preschool age [26]. This emphasizes the social aspect of obesity and the importance of considering the entire family for long-term changes [27]. Moreover, parents’ self-weight perception is crucial in motivating lifestyle changes, as they play a fundamental role in shaping children’s routines and habits. In this sense, perceiving their body weight as unhealthy does not necessarily mean they are ready to take action and make changes in the short term. Rhee and colleagues found that parents who believed their weight was above average were more likely to be in the contemplation stage rather than the action stage for weight change (OR: 7.39 vs. 3.45, respectively) [23]. Moreover, they may believe that excessive weight in the family is inherited and thus unchangeable [5]. This highlights the importance of considering parents’ motivations and previous experiences with diet and physical activity programs when planning multi-component healthy lifestyle interventions, as these experiences may condition their attitude to change [23]. The underestimation of weight status has been linked to feeding behavior in young children [19] and unhealthy attitudes toward eating habits [28]. In addition, mothers of girls with overweight may have a more negative attitude, being more likely to encourage dieting to maintain an appropriate weight [29]. Moreover, a recent study reported no associations between maternal concern and perception of their child’s overweight risk and maternal feeding practices [30]. Therefore, identifying this group of parents is crucial for providing them with support and guidance on healthy lifestyles. Accurate recognition of a child’s weight status encourages parents to adopt positive lifestyle changes [31]. Consequently, school doctors in Austria could serve as mediators between families and the health system, fostering a trusting relationship and considering each school’s social and cultural particularities that could impact weight status [3,22].

Overall, our study highlights the importance of addressing parental misperceptions about children’s weight, which is crucial as the first step for recognizing excessive weight as a health issue requiring action. Moreover, further evaluation is needed to understand how best to approach parents of children with overweight and obesity to promote action [27], as it is a sensitive topic. Parents who are informed about the negative impact of excessive body fat are more likely to make positive changes for their family’s well-being.

Some limitations of our study should be addressed. First, the non-representative sample, which may not generalize to the entire third grade population in Vienna, and the assessment of parental perception without distinguishing between mothers and fathers. However, a recent European study showed that fathers’ perceptions strongly aligned with mothers’ [20]. As previously described, the written scale used to assess parental perception might also have contributed to underestimation bias. Future studies should consider conducting interviews for more precise insights into parents’ perceptions [5]. Another limitation concerned missing data, as 6.5% of participants were excluded due to not reporting their child’s weight status perception. This could also apply to parents’ reported weight and height, accounting for missing data. Moreover, parental weight and height based on self-reporting could have introduced bias and led to the misclassification of nutritional status.

The strengths of our study include standardized data collection, objective body composition measurements in children, and the inclusion of three school samples from different Vienna neighborhoods, which were considered potential influencing factors in the analysis.

## 5. Conclusions

In conclusion, the parental underestimation of children’s weight status was more common for children with overweight or obesity than for those with normal weight in a sample of school-aged children from Vienna. As healthy habits are established early in childhood, parental involvement in obesity prevention strategies is crucial. However, our findings should be interpreted cautiously due to the limited sample size covering only three schools and the high percentage (61.1%) of families with an immigrant background, which may only partially represent the broader population. This suggests the need for future studies to include a more diverse population within Vienna and Austria. Health professionals, especially school doctors in Austria, should screen for parental perception of weight status as a first step in identifying at-risk groups. The EDDY program now has the opportunity to add content related to parental weight status perception to its tailored strategies to prevent childhood obesity.

## Figures and Tables

**Table 1:** General characteristics of participants.

Variable	All (n = 128)	Female (n = 51)	Male (n = 77)	*p*-Value
Age (years)	7.9 (7.2–9.3)	7.9 (7.2–8.8)	7.9 (7.3–9.6)	0.19
SDS-BMI	0.28 (−1.25–2.71)	0.39 (−1.00–2.83)	0.21 (−1.39–2.47)	0.37
BMI (kg/m^2^)	16.5 (14.1–25.2)	16.8 (14.0–26.3)	16.3 (14.1–23.7)	0.43
BMI classification, N (%)				
low weight	1 (0.8)	0 (0)	1 (1.3)	0.011
normal weight	92 (71.9)	37 (72.6)	55 (71.4)	
overweight	16 (12.5)	2 (3.9)	14 (18.2)	
obesity	19 (14.8)	12 (23.5)	7 (9.1)	
Parents perception weight status, N (%)				
underweight	34 (26.6)	10 (19.6)	24 (31.2)	0.33
the right weight	76 (59.4)	31 (60.8)	45 (58.4)	
a bit overweight	16 (12.5)	9 (17.7)	7 (9.1)	
very overweight	2 (1.5)	1 (1.9)	1 (1.3)	
Maternal age				
≤37 years	62 (52.1)	23 (50.0)	39 (53.4)	0.72
≥38 years	57 (47.9)	23 (50.0)	34 (46.6)	
Mother’s BMI, (kg/m^2^) ^1^	24.8 (20.0–36.8)	25.3 (19.8–36.8)	24.1 (20.0–36.5)	0.06
Father’s BMI, (kg/m^2^) ^1^	27.7 (21.3–36.3)	28.3 (21.8–36.3)	26.9 (20.8–35.0)	0.36
Maternal nutritional status, N (%)				
Non-excessive weight	63 (51.6)	20 (40.8)	43 (58.9)	0.05
Excessive weight	59 (48.4)	29 (59.2)	30 (41.1)	
Parental background ^2^, N (%)				
one parent from Austria	21 (16.7)	8 (16.0)	13 (17.1)	0.35
both parents from Austria	28 (22.2)	8 (16.0)	20 (26.3)	
no parent from Austria	77 (61.1)	34 (68.0)	43 (56.6)	
Household income ^3^, N (%)				
<1500 €	15 (12.8)	7 (14.6)	8 (11.6)	0.14
≥1500 <2500 €	36 (30.8)	11 (22.9)	25 (36.2)	
≥2500 <5000 €	56 (47.8)	28 (58.3)	28 (40.6)	
≥5000 €	10 (8.6)	2 (4.2)	8 (11.6)	
Number of siblings, N (%)				
0	19 (14.8)	8 (15.7)	11 (14.3)	0.90
1–2	84 (65.6)	34 (66.7)	50 (64.9)	
3 or more	25 (19.5)	9 (17.6)	16 (20.8)	
Mother’s education ^4^, N (%)				
≤4 years	17 (13.6)	8 (16.0)	9 (12.0)	0.26
5–9 years	17 (13.6)	5 (10.0)	12 (16.0)	
10–13 years	65 (52.0)	30 (60.0)	35 (46.7)	
≥13 years	26 (20.8)	7 (14.0)	19 (25.3)	
Father’s education ^5^, N (%)				
≤4 years	14 (11.7)	6 (12.8)	8 (11.0)	0.96
5–9 years	14 (11.7)	6 (12.8)	8 (11.0)	
10–13 years	72 (60.0)	28 (59.5)	44 (60.2)	
≥13 years	20 (16.6)	7 (14.9)	13 (17.8)	

^1^ By parental report on weight and height, based on data from n = 123 mothers and n = 114 fathers. ^2^ As assessed by parents’ nationality (n = 126). ^3^ Based on report of all family members’ income, including family allowance (n = 117). ^4^ Based on n = 125 answers. ^5^ Based on n = 120 answers.

**Table 2:** Characteristics by parental perception of their child’s weight status.

	Parental Misperception		
	Underestimation	Agreement	Overestimation
N (%)	62 (48.4)	64 (50)	2 (1.6)
Sex, N (%)			
Female	23 (37.1)	26 (40.6)	2 (100)
Male	39 (62.9)	38 (59.4)	0 (0)
Age (years)	7.9 (7.3–9.6)	7.9 (7.2–9.1)	7.5 (7.1–8.0)
SDS-BMI	0.58 (−1.27–2.77)	0.26 (−1.06–1.81)	0.79 (0.57–1.01)
BMI (kg/m^2^)	16.9 (14.0–26.3)	16.3 (14.1–20.9)	17.5 (16.8–18.1)
Child nutritional status, N (%)			
Non-excessive weight	34 (54.8)	57 (89.1)	2 (100)
Excessive weight	28 (45.2)	7 (10.9)	0 (0)
Mother’s age (years)	36 (26–44)	38 (29–48)	37 (35–39)
Father’s age (years)	39 (29–52)	39 (32–51)	37 (35–39)
Maternal age, N (%)			
≤37 years	31 (56.4)	30 (48.4)	1 (50.0)
≥38 years	24 (43.6)	32 (51.6)	1 (50.0)
Mother’s BMI, (kg/m^2^) ^1^	26.4 (19.4–37.6)	23.3 (20.2–36.1)	22.3 (19.4–25.1)
Father’s BMI, (kg/m^2^) ^1^	28.0 (20.6–37.5)	26.9 (21.8–33.5)	27.8 (23.0–32.6)
Maternal nutritional status, N (%)			
Non-excessive weight	24 (40.7)	38 (62.3)	1 (50.0)
Excessive weight	35 (59.3)	23 (37.7)	1 (50.0)
Parental background ^2^, N (%)			
one parent from Austria	11 (18.3)	9 (14.1)	1 (50.0)
both parents from Austria	14 (23.3)	13 (20.3)	1 (50.0)
no parent from Austria	35 (58.3)	42 (65.6)	0 (0)
Household income ^3^, N (%)			
<1500 €	10 (17.9)	5 (8.5)	0 (0)
≥1500 <2500 €	19 (33.9)	17 (28.8)	0 (0)
≥2500 <5000 €	24 (42.9)	30 (50.9)	2 (100.0)
≥5000 €	3 (5.4)	7 (11.9)	0 (0)
Number of siblings, N (%)			
0	9 (14.5)	9 (14.1)	1 (50.0)
1–2	42 (67.7)	41 (64.1)	1 (50.0)
3 or more	11 (17.7)	14 (21.9)	0 (0)
Maternal education ^4^, N (%)			
<13 years	50 (83.3)	47 (74.6)	2 (100.0)
≥13 years	10 (16.7)	16 (25.4)	0 (0)

^1^ By parental report on weight and height, based on data from n = 123 mothers and n = 114 fathers. ^2^ As assessed by parents’ nationality (n = 126). ^3^ Based on report of all family members’ income, including family allowance (n = 117). ^4^ Based on n = 125 answers.

**Table 3:** Unadjusted and adjusted odds ratios for underestimation of child’s weight status according to associated factors.

	Parental Underestimation of Body Weight	
	OR (95% CI)	aOR (95% CI)
Sex		
Male	1.25 (0.61–2.54)	1.71 (0.71–4.13)
Female	1	1
Maternal nutritional status		
Non-excessive weight	0.42 (0.20–0.87) *	0.74 (0.32–1.74)
Excessive weight	1	1
Child nutritional status		
Non-excessive weight	0.14 (0.06–0.37) ^ǂ^	0.16 (0.050–0.49) ^§^
Excessive weight	1	1
Household income ^1^		
<1500 €	4.66 (0.83–26.2)	8.23 (0.96–70.6)
≥1500 <2500 €	2.61 (0.58–11.7)	3.24 (0.53–19.8)
≥2500 <5000 €	1.75 (0.41–7.47)	2.44 (0.42–14.0)
≥5000 €	1	1
Number of siblings		
0	1.15 (0.35–3.79)	1.31 (0.32–5.47)
1–2	1.27 (0.52–3.12)	1.24 (0.43–3.59)
3 or more	1	1
Maternal education ^2^		
<13 years	1.63 (0.68–3.95)	1.33 (0.45–4.00)
≥13 years	1	1

OR: odds ratios. CI: Confidence interval. aOR: adjusted OR, with odds ratios adjusted by logistic regression for maternal nutritional status, child’s nutritional status, household income and school used as control variables in the model. ^1^ Based on report of all family members’ income, including family allowance (n = 117). ^2^ Based on n = 125 answers. ^ǂ^ *p* < 0.0001; ^§^ *p* < 0.002; * *p* < 0.05.

## Data Availability

The data presented in this study are available on request from the corresponding author due to ethical reasons.

## Supplementary Materials

The following supporting information can be downloaded at: https://www.mdpi.com/article/10.3390/nu16234094/s1, Figure S1: Correlation plot between children and mother’s body mass index (BMI).
