# Supplementary material for: How Parents Perceive Their Children’s Body Weight: Insights from a Sample of Schoolchildren from Vienna, Austria
Source: Nutrients. 2024 Nov 27;16(23):4094. doi: 10.3390/nu16234094 (PMC11643454; doi:10.3390/nu16234094)
Supplement: Supplementary file 1 [file nutrients-16-04094-s001.zip › nutrients-3337080-supplementary.pdf]

Article

How parents perceive their children's body weight: insights from a sample of school-children from Vienna, Austria

Supplementary Materials

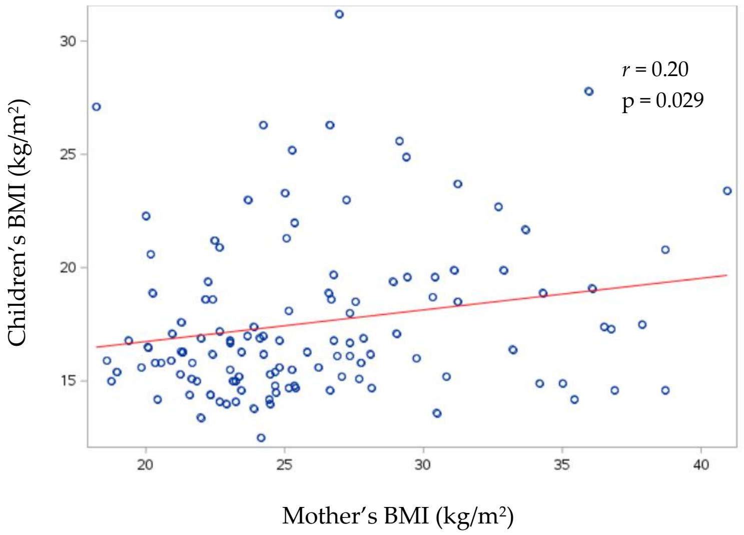

Figure S1. Correlation plot between children and mother's body mass index (BMI).
